# Prognostic significance of S100A4 expression in stage II and III colorectal cancer: results from a population‐based series and a randomized phase III study on adjuvant chemotherapy

**DOI:** 10.1002/cam4.766

**Published:** 2016-06-08

**Authors:** Kjetil Boye, Havjin Jacob, Kari‐Anne M. Frikstad, Jahn M. Nesland, Gunhild M. Mælandsmo, Olav Dahl, Arild Nesbakken, Kjersti Flatmark

**Affiliations:** ^1^ Department of Tumor BiologyInstitute for Cancer ResearchNorwegian Radium Hospital, Oslo University HospitalOsloNorway; ^2^ Department of OncologyNorwegian Radium Hospital, Oslo University HospitalOsloNorway; ^3^ Section for OncologyDepartment of Clinical ScienceFaculty of Medicine and DentistryUniversity of BergenBergenNorway; ^4^ Department of PathologyNorwegian Radium Hospital, Oslo University HospitalOsloNorway; ^5^ Medical FacultyUniversity of OsloOsloNorway; ^6^ Department of PharmacyFaculty of Health SciencesUniversity of TromsøTromsøNorway; ^7^ Department of Oncology and RadiophysicsHaukeland University HospitalBergenNorway; ^8^ K.G. Jebsen Colorectal Cancer Research CentreOslo University HospitalOsloNorway; ^9^ Centre for Cancer BiomedicineFaculty of MedicineUniversity of OsloOsloNorway; ^10^ Department of Gastroenterological SurgeryUllevål Hospital, Oslo University HospitalOsloNorway; ^11^ Department of Gastroenterological SurgeryNorwegian Radium Hospital, Oslo University HospitalOsloNorway

**Keywords:** 5‐fluorouracil, adjuvant chemotherapy, colorectal cancer, prognostic factor, S100A4

## Abstract

Current clinical algorithms are unable to precisely predict which colorectal cancer patients would benefit from adjuvant chemotherapy, and there is a need for novel biomarkers to improve the selection of patients. The metastasis‐promoting protein S100A4 predicts poor outcome in colorectal cancer, but whether it could be used to guide clinical decision making remains to be resolved. S100A4 expression was analyzed by immunohistochemistry in primary colorectal carcinomas from a consecutively collected, population‐representative cohort and a randomized phase III study on adjuvant 5‐fluorouracil/levamisole. Sensitivity to treatment with 5‐fluorouracil in S100A4 knockdown cells was investigated using 2D and 3D cell culture assays. Strong nuclear expression of S100A4 was detected in 19% and 23% of the tumors in the two study cohorts, respectively. In both cohorts, nuclear immunoreactivity was associated with reduced relapse‐free (*P *<* *0.001 and *P *=* *0.010) and overall survival (*P *=* *0.046 and *P *=* *0.006) in univariate analysis. In multivariate analysis, nuclear S100A4 was a predictor of poor relapse‐free survival in the consecutive series (*P *=* *0.002; HR 1.9), but not in the randomized study. Sensitivity to treatment with 5‐fluorouracil was not affected by S100A4 expression in in vitro cell culture assays, and there was no indication from subgroup analyses in the randomized study that S100A4 expression was associated with increased benefit of adjuvant treatment with 5‐fluorouracil/levamisole. The present study confirms that nuclear S100A4 expression is a negative prognostic biomarker in colorectal cancer, but the clinical utility in selection of patients for adjuvant fluoropyrimidine‐based chemotherapy is limited.

## Introduction

Colorectal cancer (CRC) is one of the most common cancer types worldwide and a major cause of cancer death. The prognosis of patients with CRC is highly dependent on the tumor stage at diagnosis. Five‐year overall survival for patients with stage I disease is approximately 90%, decreasing to about 10% for stage IV patients 1. In curatively resected CRC, certain stage‐specific factors provide additional prognostic value, such as tumor perforation, T4 status, and number of examined lymph nodes in stage II and the number of involved lymph nodes in stage III 1, 2. Still, the biological behavior of the tumors within each tumor stage varies considerably, and a more precise prediction of patient outcome could lead to better treatment decisions.

Adjuvant fluoropyrimidine‐based chemotherapy is routinely offered to colon cancer patients in TNM stage III and to selected high‐risk stage II patients. The absolute survival advantage in stage III is approximately 10–15%, whereas a clear difference in overall survival has not been shown in stage II 2. Consequently, the majority of patients receiving adjuvant chemotherapy do not benefit from the treatment, either because they would have been cured by surgery alone or because they nevertheless experience disease recurrence. Thus, new prognostic and predictive factors to improve the selection of patients for adjuvant treatment would be of great clinical importance.

S100A4 is a small, multifunctional, Ca^2+^‐binding protein with the ability to promote invasion and metastasis in several cancer types 3. In CRC, S100A4 is overexpressed compared to normal mucosa and adenomas, and higher expression has been observed in liver metastases than in primary tumors 4, 5. Several studies have shown that S100A4 is a strong predictor of poor survival in patients undergoing curatively intended surgery 6, 7, 8, 9, 10, 11, 12. The protein is localized in the nucleus and the cytoplasm, and nuclear expression seems to be the best prognostic parameter 6, 13. Suggested biological functions of nuclear S100A4 include interaction with p53 and inhibition of p53‐dependent cell cycle arrest and apoptosis 14 and colocalization with centrosomes and regulation of the G2/M phase 15, but the phenotypic outcome of nuclear S100A4 expression is in general incompletely characterized. Even though there is a large body of evidence documenting the prognostic impact of S100A4 in CRC, it remains to be resolved whether immunohistochemical staining for S100A4 could be an adjunct to other biomarkers in routine clinical practice. Here, we have investigated the clinical impact of S100A4 expression in a large, unselected, population‐representative cohort and in a randomized study on adjuvant chemotherapy in stage II and III patients.

## Materials and Methods

### Patient cohorts

Study cohort 1 is a population‐representative consecutive series of CRC patients from Oslo University Hospital Aker, and has been previously reported 16, 17, 18. Briefly, all patients with CRC from 1993 to 2003 were registered and clinical data were prospectively recorded. Of the 1290 patients included, 929 underwent a major resection and had tumor samples available for tissue microarray (TMA) construction 17. Colon cancer patients ≤75 years of age and all rectal cancer patients who underwent curative surgery entered a 5‐year follow‐up program as described previously 18. Study cohort 2 consists of CRC patients included in a Norwegian randomized phase III study on adjuvant 5‐fluorouracil/levamisole 19. Four hundred twenty‐five patients who had a radical resection for an adenocarcinoma of the colon or rectum were included in the study between January 1993 and October 1996. Of the 412 evaluable patients, 206 patients were randomized to 49 weeks of 5‐fluorouracil and levamisole and 206 patients to no further treatment after radical surgery. Both studies were approved by the Regional Ethics' Committees and written informed consent was obtained.

### Immunohistochemistry

The construction of TMAs has been described previously 17, 20. Sections from the TMAs were immunostained using the Dako EnVision Flex+ detections system (Dako, Glostrup, Denmark) as described previously 21. The monoclonal anti‐S100A4 antibody 20.1 in a final concentration of 1.8 μg/mL was used 22. Negative controls included replacement of the primary antibody with mouse myeloma protein of the same subclass and concentration, and preabsorption experiments using recombinant S100A4. Sections from CRC tumor tissue known to express high levels of S100A4 were used as positive controls.

### Evaluation of immunohistochemistry

For study cohort 1, S100A4 immunoreactivity was evaluated independently by two of the authors (K. B. and K. F.), and nuclear and cytoplasmic staining were registered as separate variables. Staining intensity was scored as negative, weak, moderate, and strong. The number of positive tumor cells was estimated and grouped as follows: 0 (0%), 1 (<1%), 2 (1–10%), 3 (11–33%), 4 (34–66%), and 5 (*>*66%). Discrepant results were reviewed in common and consensus was reached. For all analyses, negative/weak and moderate/strong staining intensity were grouped and considered negative and positive, respectively, and only cases with moderate or strong intensity (defined as positive) were classified with respect to the fraction of positive tumor cells. Based on the number of cases in the percentage groups, group 1–4 (1–66%) was considered moderately positive and group 5 (>66%) strongly positive. For study cohort 2, immunoreactivity was scored as negative, moderate, and strong according to the criteria defined through analysis of cohort 1, and dichotomized as negative/moderate and strong for all analyses. Evaluation was performed by two independent observers (H. J. and J. M. N.).

### Statistical analysis

Associations between S100A4 staining and clinicopathological variables were tested using Fisher's exact test, linear‐by‐linear association chi‐square test, or independent samples *t* test as appropriate. Univariate survival analysis was performed according to the Kaplan–Meier method, and survival was compared using the log rank test. Multivariate analysis was conducted using the Cox proportional hazards regression model with backward, stepwise elimination of variables. The time from surgery (cohort 1) or randomization (cohort 2) to diagnosis of distant or local recurrence was recorded as relapse‐free survival. Patients were censored at death of any cause or when lost to follow‐up. Overall survival was defined as the time from surgery or randomization to death of any cause. Median follow‐up for overall survival for patients still alive was 9.7 years for cohort 1 (range 5.2–17.3) and 7.6 years (range 4.9–11.0) for cohort 2. Data analysis was performed using SPSS version 21.0 (SPSS Inc., Chicago, IL, USA). All *P* values are two‐tailed and considered significant when *P *<* *0.05.

### Cell culture and treatment

The human CRC cell lines HCT116 and SW620 were obtained from American Type Cell Collection (ATCC; Manassas, VA). Cells were cultured in RPMI 1640 (Lonza, Basel, Switzerland) supplemented with 8% (v/v) fetal bovine serum (FBS; Life Technologies, Carlsbad, CA), 1% (v/v) Hepes buffer (Lonza), and 1% (v/v) Glutamax (Life Technologies). The generation of stably transduced cells expressing shRNA against S100A4 (shA4) has been described previously 15. Cell line identity was validated by short tandem repeat analysis using the STR PowerPlex16 System (Promega, Fitchburg, WI), and all cell cultures were routinely tested for *Mycoplasma* infection. Prior to experiments, subconfluent cells were trypsinated (Lonza), seeded in multiwell dishes or cell culture flasks, and allowed to adhere overnight. The cell culture medium was then removed and replaced with fresh culture medium with or without 5‐fluorouracil (5FU; Hospira, Lake Fores, IL) as indicated. The cells were incubated for 4 h before the drug was removed, fresh medium was added to each well, and cells were further incubated for the time periods indicated before harvesting.

### Cell viability

Cells were seeded at a density of 1.5 × 10^4^ (HCT116) or 2.25 × 10^4^ (SW620) cells/cm^2^ in 96‐well plates, treated as described earlier, and incubated for 72 h after removal of drug. Cell viability was measured using CellTiter 96^®^ AQ_ueous_ One Solution Cell Proliferation Assay (Promega) according to the manufacturer's instructions.

### Clonogenic survival

One hundred cells per well were seeded in a volume of 2 mL in 6‐well plates and treated as described earlier. The plates were incubated for about 1 week until colonies of appropriate size were detectable by visual inspection. The colonies were fixed using ice‐cold methanol for 1 min at room temperature and stained with 0.05% crystal violet dye (Apotekproduksjon, Oslo, Norway). Colonies containing ≥50 tumor cells were then counted manually at a Gerber Counter (Gerber Scientific Products, Tolland, CT).

### Spheroid assay

The protocols for forming spheroids and measuring cell viability by calculated spheroid volume were adapted from Vinci and coworkers 23. Briefly, 1000 cells per well were seeded in 96‐well, ultralow attachment plates (Costar, Washington, DC) and incubated for 72 h. The spheroids were then treated with chemotherapy as described earlier. The spheroids were photographed using an IX81‐motorized inverted microscope (Olympus, Shinjuku, Tokyo, Japan) 96 h after treatment, and the software Cell^P (Olympus) was used to calculate the spheroid volume. The average radius of the spheroids was calculated, and spheroid volumes computed with the formula V = 4/3πr^3^, based on the assumption that the spheroids were approximately spherical.

### Protein analysis

Protein isolation and immunoblotting was performed as described previously 15.

## Results

### Study cohort 1

**Figure 1:**
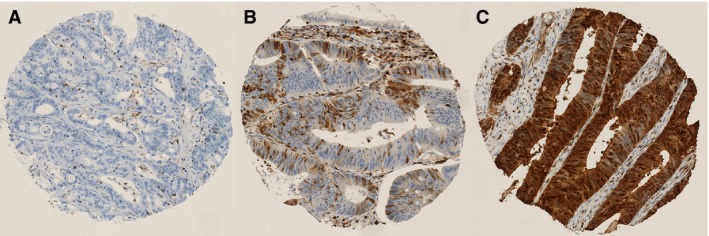
Representative photomicrographs showing immunohistochemical staining of S100A4. (A) No immunoreactivity in the cytoplasm and nucleus. (B) Moderately positive nuclear and cytoplasmic staining. (C) Strong nuclear and cytoplasmic staining.

The clinical and histopathological data of the study cohort are presented in Table 1. Median age was 73 years (range 29–94). Of the 929 tumor samples present on the TMA, 146 cases were not evaluable for technical reasons or because no tumor tissue was present, leaving 783 cases available for S100A4 analysis. In 64 cases there were discrepant results in scoring of nuclear immunoreactivity between the two observers, resulting in a κ value of 0.73 (classified as “substantial agreement”). Nineteen percent of the tumors displayed strong nuclear S100A4 expression, while 44% showed strong cytoplasmic staining (Table 2; complete scoring results are presented in Table S1). Representative photomicrographs of immunohistochemical staining of S100A4 are presented in Figure 1. Associations between S100A4 expression and clinical and histopathological characteristics are shown in Table S2. There was a weak relationship between S100A4 nuclear staining and male gender, advanced T stage, and poor tumor differentiation. No other statistically significant associations were discovered.

**Table 1:** Baseline clinical and histopathological data of study cohorts 1 and 2

Parameters	Study cohort 1^a^		Study cohort 2^a^	
	*N*	%	*N*	%
Gender				
Female	415	53	179	48
Male	368	47	191	52
TNM stage				
I	119	15	0	0
II	317	41	219	59
III	198	25	151	41
IV	148	19	0	0
pT				
1	30	4	3	1
2	111	14	28	8
3	561	72	316	85
4	81	10	23	6
pN				
0	483	62	219	59
1	212	27	100	27
2	81	10	51	14
ND	7		0	
Differentiation				
Well/moderate	663	88	306	84
Poor	93	12	58	16
ND	27		6	
Tumor localization				
Colon	589	75	261	71
Rectum	189	24	109	29
Synchronous	5	1	0	0

ND, not determined.

a A total of 783 and 370 patients were examined.

**Table 2:** Immunohistochemical expression of S100A4 in study cohorts 1 and 2

	Nuclear staining^a^	Cytoplasmic staining^a^
Study cohort 1		
Negative/moderate	635 (81)	439 (56)
Strong	148 (19)	344 (44)
Study cohort 2		
Negative/moderate	286 (77)	286 (77)
Strong	84 (23)	84 (23)

a The number of cases and percentages (in parentheses) are shown.

**Figure 2:**
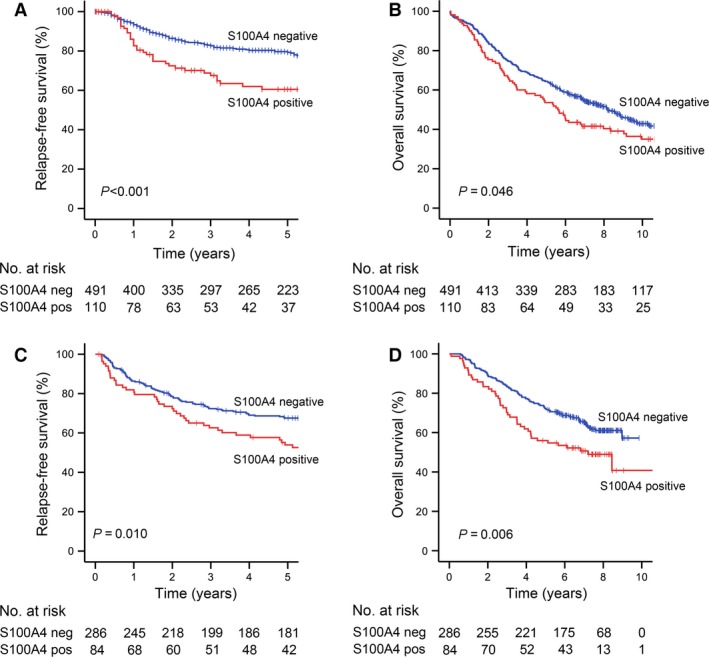
Kaplan–Meier survival curves based on the expression of nuclear S100A4. (A) Relapse‐free survival and (B) overall survival in study cohort 1. (C) Relapse‐free survival and (D) overall survival in study cohort 2.

For the survival analyses, patients with TNM stage IV (*n* = 148), unknown TNM stage (*n* = 1), or R1/R2 resection (*n* = 33) were excluded, resulting in an outcome cohort of 601 patients. Of these, 116 (19%) experienced a recurrence during follow‐up. Ninety patients were registered with distant metastasis, 12 with isolated local recurrence, and 14 with local and distant recurrence. Strong nuclear expression of S100A4 was a powerful prognostic factor for reduced relapse‐free survival in univariate analysis, with an estimated 5‐year survival of 61% compared to 80% for negative and moderate staining (*P *<* *0.001; Fig. 2). Patients with negative and moderate S100A4 expression had a similar outcome (Fig. S1A; *P *=* *0.20 in pairwise comparison), thus for all analyses expression was dichotomized into negative/moderate and strong. Overall survival was also significantly poorer for patients with S100A4‐positive tumors (*P *=* *0.046; Fig. 2B). In subgroup analyses, S100A4 was of prognostic significance in TNM stages II and III (Fig. S1C and D) and for patients with colon cancer and rectal cancer when analyzed separately (Fig. S1E and F). Cytoplasmic expression was not associated with relapse‐free or overall survival (Fig. S2A and B). In multivariate analysis, nuclear S100A4 was significantly associated with relapse‐free survival (*P *=* *0.002; HR 1.9; 95% CI: 1.3–2.9; Table 3). TNM stage and age were also included in the final model, and the variables entered were S100A4, TNM stage, age, gender, tumor localization (colon vs. rectum), and differentiation. Taken together, these results convincingly confirm that nuclear expression of S100A4 is a strong prognostic factor in CRC, and that the prognostic significance is retained across tumor stage and localization.

**Table 3:** Multivariate Cox regression analysis of relapse‐free survival^a^

	*P* value	Hazard ratio	95% CI
Study cohort 1			
S100A4	0.002		
Negative		Ref	
Positive		1.9	1.3–2.9
TNM stage	<0.001		
I		Ref	
II		2.1	1.0–4.2
III		5.3	2.6–10.7
Age	0.02	1.02	1.00–1.03
Study cohort 2			
TNM stage	<0.001		
II		Ref	
III		4.1	2.8–5.9
Tumor differentiation	0.03		
High/moderate		Ref	
Low		1.6	1.0–2.5

a All parameters included in the final models are shown.

### Study cohort 2

To examine the prognostic impact of S100A4 in patients receiving adjuvant treatment, we investigated the expression in tumor samples from patients included in a phase III randomized study that compared surgery alone with surgery and 5‐fluorouracil/levamisole in stage II or III colon and rectal cancer patients 19. Samples for TMA construction were available for 409 of the 412 patients 20. In 39 cases, S100A4 staining was not technically successful, leaving a study population of 370 patients. The clinical and histopathological characteristics are presented in Table 1. Median age was 63 years (range 28–75).

S100A4 expression was dichotomized as strongly positive or negative/moderate according to the criteria defined for cohort 1. In 14 of 370 cases, the observers were not in agreement with the scoring of nuclear S100A4 staining, resulting in a κ value of 0.89 (classified as “almost perfect agreement”). Eighty‐four (23%) of 370 samples were classified as strongly positive for nuclear and cytoplasmic immunoreactivity (Table 2), of which 73 cases showed strong staining in both subcellular compartments. Associations between S100A4 expression and clinicopathological parameters are shown in Table S3. Thirty‐one percent of stage III tumors displayed S100A4 nuclear expression compared to only 16% for stage II (Table S3; *P *=* *0.001). No other statistically significant associations were found.

One hundred thirty‐two of the 370 patients suffered from disease relapse (local recurrence and/or distant metastasis) during follow‐up, and 148 were registered as dead. In univariate analyses, S100A4 nuclear expression was associated with reduced relapse‐free survival (*P *=* *0.010; Fig. 2C) and overall survival (*P *=* *0.006; Fig. 2D), and cytoplasmic expression was also a predictor of poor outcome (Fig. S2C and D). Nuclear S100A4 was, however, not significantly associated with relapse‐free survival in multivariate analysis including the covariates S100A4, gender, age, TNM stage, tumor localization (colon vs. rectum), and tumor differentiation (Table 3).

### S100A4 expression and adjuvant treatment

**Figure 3:**
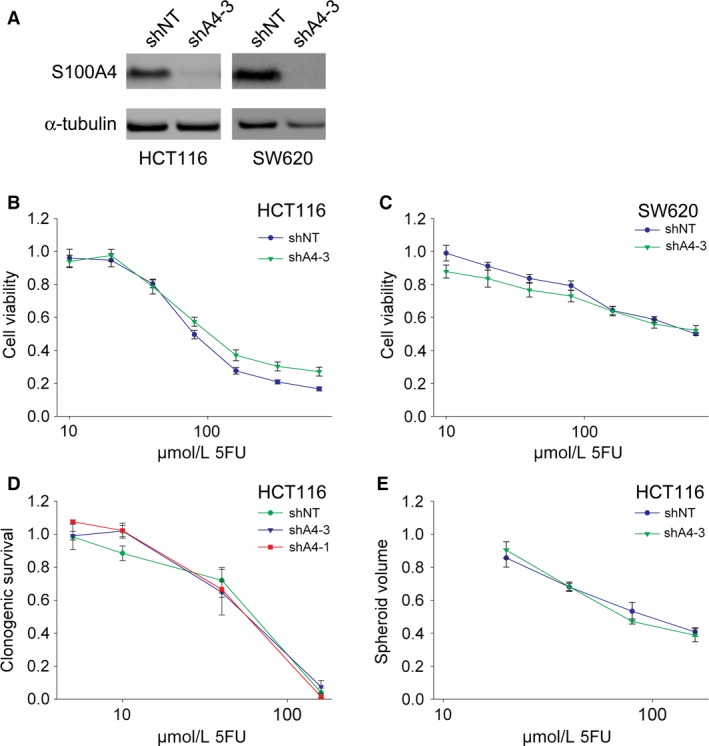
Treatment with 5‐fluorouracil in CRC cell lines stably transduced with S100A4 shRNA. (A) Immunoblots of total cell lysates from HCT116 and SW620 cells stably transduced with nontarget shRNA (shNT) or shRNA against S100A4 (shA4‐3) as indicated. Cell viability assay using HCT116 (B) and SW620 (C) cells stably transduced with nontarget shRNA (shNT) or shRNA against S100A4 (shA4‐3). Cells were treated with seven different concentrations of 5‐fluorouracil as indicated. Clonogenic survival assay (D) and spheroid assay (E) using HCT116 cells stably transduced with nontarget shRNA (shNT) or two different shRNAs against S100A4 (shA4‐1 and shA4‐3). Cells were treated with four different concentrations of 5‐fluorouracil as indicated. Cell viability, clonogenic survival, and spheroid volume are all displayed as a ratio compared to cells incubated without drug. Bars represent ± standard error of the mean. CRC, colorectal cancer.

To analyze the implication of S100A4 expression in patients receiving adjuvant treatment, we first considered TNM stage III colon cancer patients, as this was the only subgroup with a survival benefit of adjuvant chemotherapy in this study 19. Adjuvant treatment seemed to be of benefit for both S100A4‐positive and ‐negative patients (Fig. S3A and B), even if the differences were not statistically significant due to a relatively small number of patients. To further explore whether S100A4 expression in tumor cells could affect sensitivity to 5‐fluorouracil, cell culture experiments using CRC cell lines with manipulated levels of S100A4 were performed. The cell lines HCT116 and SW620 express high amounts of S100A4, and S100A4 knockdown cells were generated by stable lentiviral transduction with S100A4 shRNA (Fig. 3A). When exposed to increasing concentrations of 5‐fluorouracil, HCT116 was clearly the more sensitive cell line, but sensitivity was independent of S100A4 expression in both cell lines (Fig. 3B and C). Additional experiments were performed in HCT116 cells using the clonogenic survival assay and in three‐dimensional spheroid cell cultures, but S100A4 expression did not influence the sensitivity to 5‐fluorouracil in any of the investigated experimental conditions (Fig. 3 D and E).

Furthermore, we asked if the prognostic value of S100A4 could be used to select colon cancer patients for adjuvant treatment, either to identify stage II patients who would benefit or to identify stage III patients with an excellent prognosis who might not profit from adjuvant chemotherapy. S100A4‐negative stage III colon cancer patients had an estimated 3‐year relapse‐free survival of 66% and 55% in cohorts 1 and 2, respectively, implying that these patients likely would benefit from additional treatment. In stage II tumors of the colon, nuclear expression was a negative prognostic marker in cohort 1 (Fig. S3C), but not in the surgery‐only group in cohort 2 (Fig. S3D). Nevertheless, S100A4‐positive stage II colon cancer patients randomized to adjuvant 5‐fluorouracil and levamisole had a poor outcome with an estimated 3‐year relapse‐free survival of 71% compared to 88% for S100A4‐negative patients (Fig. S3E).

## Discussion

The findings in our study were based on two patient cohorts. The first is an unselected, consecutive series from one Norwegian hospital from 1993 to 2003 18. The series can be considered population‐based because patients were consecutively enrolled from a geographically defined catchment area, and because the completeness of the inclusion has been verified against the Cancer Registry of Norway. Less than 10% of the patients received adjuvant treatment 18, and the cohort is thus suited for investigation of prognostic markers. Furthermore, the cohort size is relatively large and allows relevant subgroup analyses, such as stratification based on TNM stage and tumor localization. The second cohort is a randomized, multicentre trial on adjuvant chemotherapy in stage II and III patients included between 1993 and 1996 19. Tumor samples for TMA construction were available from nearly all patients, and the cohort is thus well fitted for studies on prognostic and predictive tissue biomarkers in patients receiving adjuvant fluoropyrimidine‐based chemotherapy. For this purpose, it is a great advantage that one randomization arm was surgery alone, allowing the comparison between patients receiving or not receiving adjuvant treatment.

We found that 19% and 23% of the cases had strong nuclear staining in cohorts 1 and 2, respectively, whereas strong cytoplasmic staining was observed in 44% and 23% of the tumors.

The reason for the difference in cytoplasmic expression is uncertain. Clinical and demographic variables were similar in the two cohorts aside from the stage distribution, but this could not explain the discrepancy as the percentage of tumors with cytoplasmic staining in stages II and III in cohort 1 was 46%. Different observers evaluated the slides in the two series, and especially intensity scoring is a subjective judgment. However, the frequency of nuclear staining was comparable, indicating that the interobserver variability is reasonably low. Differences in TMA construction between the two cohorts might also contribute as one 0.6 mm core per tumor was used for study cohort 1 compared to one to three cylinders for cohort 2. The percentage of S100A4‐positive tumors in previous studies in CRC has varied considerably due to different scoring algorithms and cutoffs, patient selection, and technical reasons (e.g., antibodies used and TMAs vs. whole sections). Here, we used a cutoff for strong staining of 66%, which is similar to the two other large series where TMAs were employed 7, 9. Gongoll and coworkers used 50% as a cutoff, but did not evaluate cytoplasmic and nuclear staining separately, whereas Kho et al. applied 50–70% based on the optimal dichotomy for prognostic significance. In our previous study using whole sections a cutoff of 1% was used 6, but the frequency of positive cells might not be comparable between TMAs and whole sections since a large degree of intratumoral heterogeneity of S100A4 expression is observed in CRC 5, 9.

Nuclear expression was a stronger predictor of poor prognosis than cytoplasmic expression in cohort 1, supporting the findings in our previous study 6. In cohort 2, S100A4 expression in both subcellular localizations had prognostic significance, which might reflect the large degree of overlap between cytoplasmic and nuclear staining in this study, as only 22 of 370 tumors displayed strong expression exclusively for either compartment. Only two previous investigations on CRC have recorded nuclear and cytoplasmic S100A4 as separate variables 6, 9, and nuclear expression was a predictor of inferior outcome in both studies, albeit not after adjustment for other prognostic variables in the Australian series. Despite differences between studies, a high level of S100A4 expression either in the nucleus or cytoplasm is associated with reduced survival in all large CRC series published. Based on the two large, unselected patient cohorts in the present report, we conclude that nuclear S100A4 is established as a biomarker of poor outcome in stage II and III CRC, and that further studies to investigate its prognostic role in this patient group are not needed.

Even though S100A4 has proven to be a robust prognostic biomarker, its utility in clinical practice is not obvious. A negative prognostic factor could be used to identify patients that would benefit from additional treatment, for example, to select stage II patients for adjuvant chemotherapy. Indeed, S100A4‐positive stage II patients had a poor prognosis in cohort 1, similar to the results in our previous report 6. In cohort 2, S100A4‐positive stage II patients randomized to surgery alone had a similar outcome as S100A4‐negative (Fig. S3D), but there were only 10 patients in the S100A4‐positive group, suggesting that the unexpectedly good outcome compared to the other cohorts might have occurred by chance. Nevertheless, there was no obvious benefit of adjuvant chemotherapy for S100A4‐positive stage II patients (Fig. S3E), indicating that the adjuvant treatment regimen used was not able to improve the inferior outcome associated with strong expression of S100A4. However, the study was not sufficiently powered to detect an effect in subgroup analyses, and the chemotherapy used is inferior to regimens with 5‐fluorouracil and oxaliplatin, which is the standard of care today. Furthermore, there were no indications from in vitro experiments that S100A4 is associated with increased sensitivity to 5‐fluorouracil. Taken together, our data indicate that S100A4 is not a useful biomarker for the selection of patients for adjuvant fluoropyrimidine‐based chemotherapy in CRC.

Still, patients with S100A4‐positive tumors fare worse than S100A4‐negative tumors, and the survival difference between the groups seems clinically relevant. S100A4‐positive stage II patients in cohort 1 have an estimated 5‐year relapse‐free survival of 66% compared to 84% for S100A4‐negative patients. The corresponding numbers for stage III patients are 46% and 64%, respectively. With such inferior relapse‐free survival rates, patients with S100A4‐expressing tumors might benefit from other systemic treatment than fluoropyrimidine‐based chemotherapy. Therapeutic approaches specifically targeting S100A4 function have been suggested 24, 25, but further development might be hampered by the lack of knowledge about the biological function of nuclear S100A4 3, 15. Another strategy could be to target the phenotype induced by S100A4 overexpression. S100A4 promotes epithelial–mesenchymal transition 3, and a mesenchymal phenotype is often less sensitive to conventional cancer drugs 26. Design of novel therapies aiming at the mesenchymal, stem‐cell‐like state might thus be beneficial also for the S100A4‐positive subpopulation of tumor cells.

In conclusion, the present study confirms that nuclear S100A4 is a negative prognostic biomarker in patients with stage II and III carcinoma of the colon or rectum. There was, however, no indication that S100A4 expression could be a predictive biomarker for response to fluoropyrimidine‐based chemotherapy. Further exploring the mechanisms involved in S100A4‐mediated disease progression could hopefully contribute to the development of new therapeutic approaches for this patient group, which certainly is in need of better treatment options.

## Conflict of Interest

None declared.

## Supporting information


**Figure S1.** Kaplan‐Meier survival curves of relapse‐free survival from study cohort 1 stratified based on expression of nuclear S100A4. (A) Data from the complete outcome cohort. (B‐D) Subgroup analyses in TNM stage I, II and II, respectively. (E, F) Subgroup analyses for patients with tumors localized in the colon and rectum, respectively.

**Figure S2.** Kaplan‐Meier survival curves based on cytoplasmic expression of S100A4. (A) Relapse‐free survival in study cohort 1. (B) Overall survival in study cohort 1. (C) Relapse‐free survival in study cohort 2. (D) Overall survival in study cohort 2.

**Figure S3.** Kaplan‐Meier survival curves of relapse‐free survival for patients with tumors localized in the colon. (A) S100A4‐negative stage III patients from study cohort 2 stratified based on randomization arm. (B) S100A4‐positive stage III patients from study cohort 2 stratified based on randomization arm. (C) Stage II patients from study cohort 1 stratified based on nuclear S100A4 expression. (D) Stage II patients from study cohort 2 randomized to surgery alone stratified based on nuclear S100A4 expression. (E) Stage II patients from study cohort 2 randomized to adjuvant chemotherapy stratified based on nuclear S100A4 expression.

**Table S1.** Complete scoring results of S100A4 immunohistochemistry in study cohort 1.
**Table S2.** Associations between S100A4 expression and clinicopathological parameters in study cohort 1.
**Table S3.** Associations between S100A4 expression and clinicopathological parameters in study cohort 2.
