# Supplementary figures and images for: Prognostic significance of S100A4 expression in stage II and III colorectal cancer: results from a population‐based series and a randomized phase III study on adjuvant chemotherapy
Source: Cancer Med. 2016 Jun 8;5(8):1840–9. doi: 10.1002/cam4.766 (PMC4971912; doi:10.1002/cam4.766)

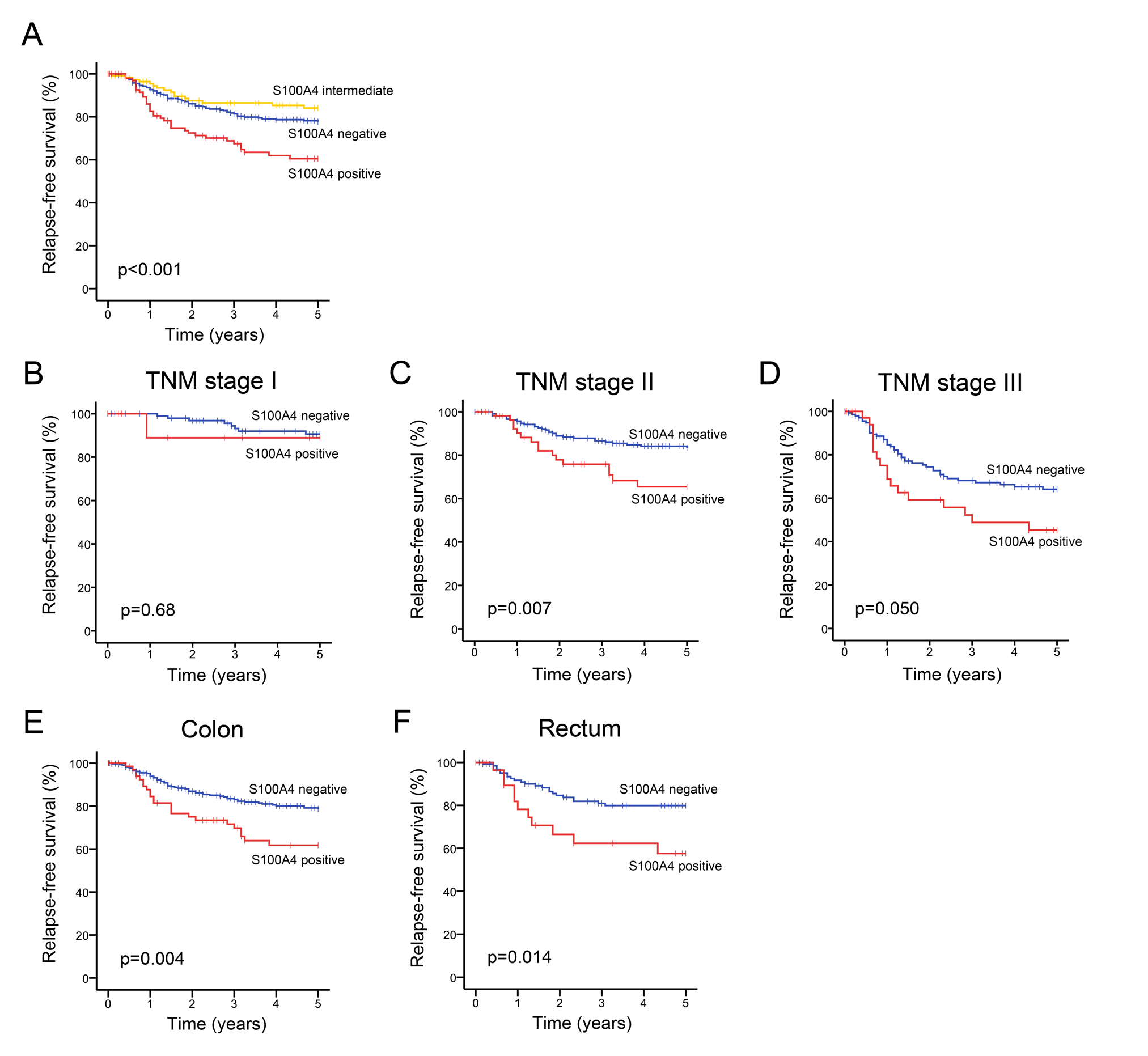

Supplement: Supplementary file 1 — Figure S1. Kaplan‐Meier survival curves of relapse‐free survival from study cohort 1 stratified based on expression of nuclear S100A4. (A) Data from the complete outcome cohort. (B‐D) Subgroup analyses in TNM stage I, II and II, respectively. (E, F) Subgroup analyses for patients with tumors localized in the colon and rectum, respectively. [file CAM4-5-1840-s001.tif]

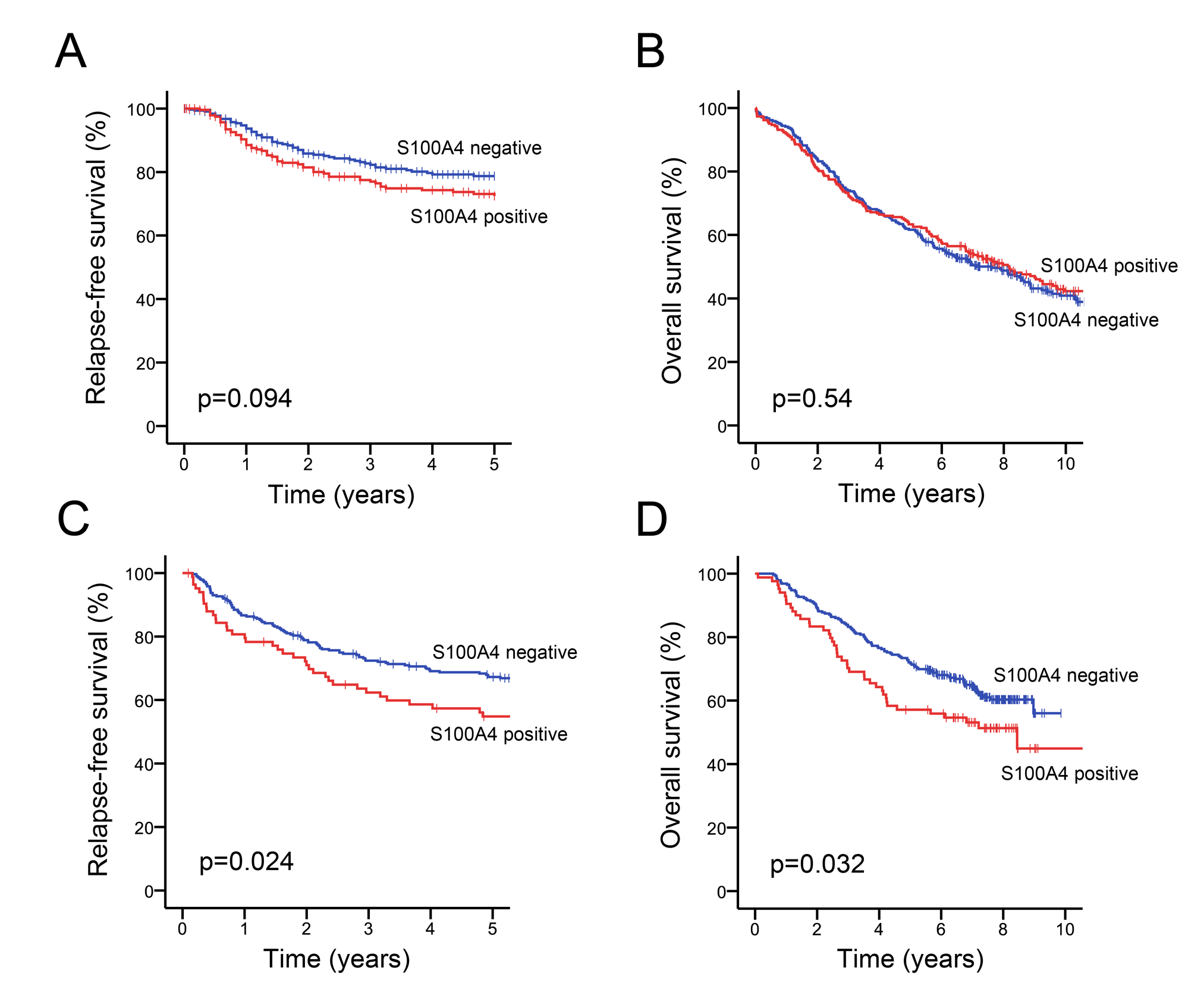

Supplement: Supplementary file 2 — Figure S2. Kaplan‐Meier survival curves based on cytoplasmic expression of S100A4. (A) Relapse‐free survival in study cohort 1. (B) Overall survival in study cohort 1. (C) Relapse‐free survival in study cohort 2. (D) Overall survival in study cohort 2. [file CAM4-5-1840-s002.tif]

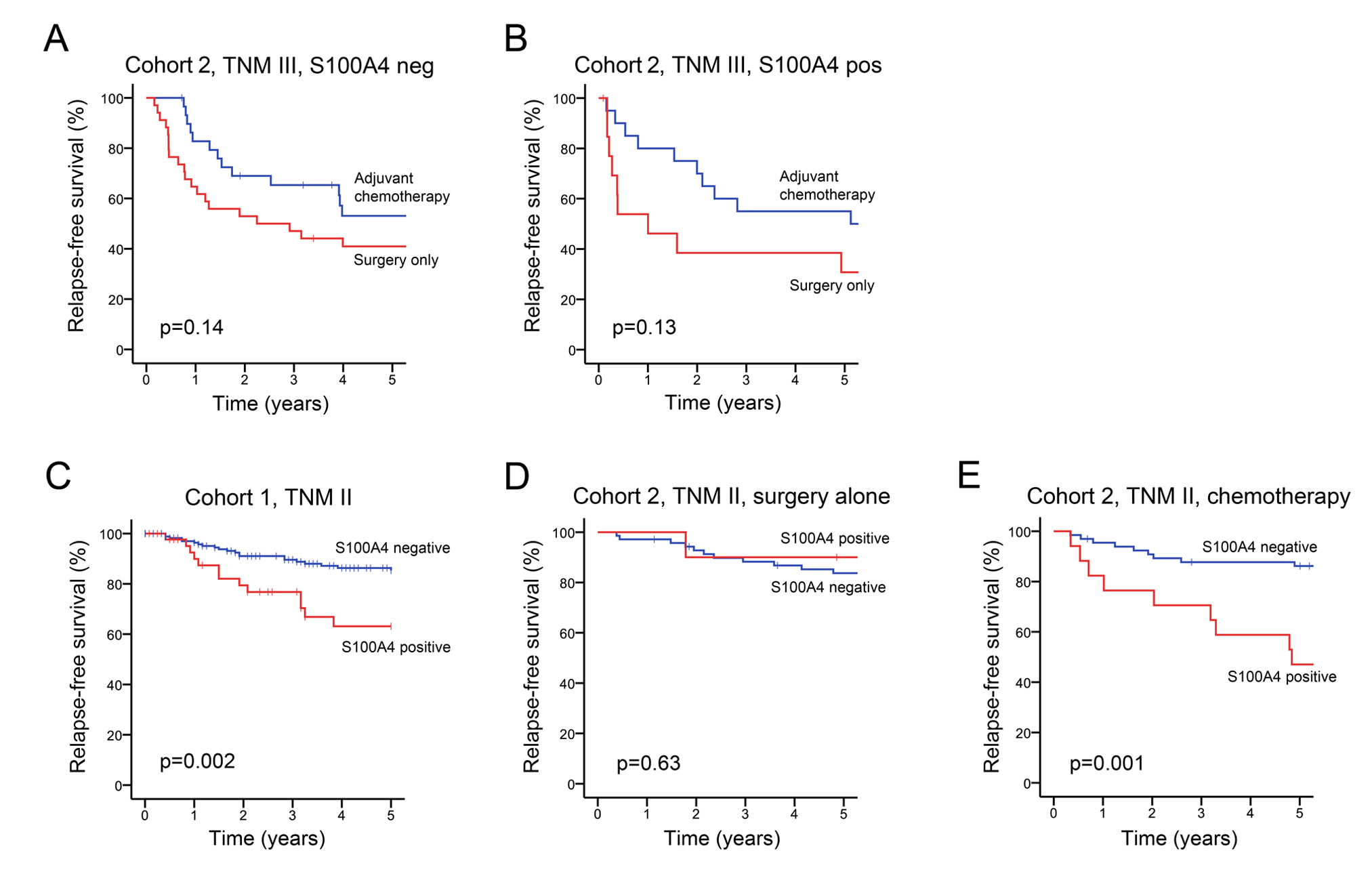

Supplement: Supplementary file 3 — Figure S3. Kaplan‐Meier survival curves of relapse‐free survival for patients with tumors localized in the colon. (A) S100A4‐negative stage III patients from study cohort 2 stratified based on randomization arm. (B) S100A4‐positive stage III patients from study cohort 2 stratified based on randomization arm. (C) Stage II patients from study cohort 1 stratified based on nuclear S100A4 expression. (D) Stage II patients from study cohort 2 randomized to surgery alone stratified based on nuclear S100A4 expression. (E) Stage II patients from study cohort 2 randomized to adjuvant chemotherapy stratified based on nuclear S100A4 expression. [file CAM4-5-1840-s003.tif]
