# Supplementary material for: Prognostic significance of S100A4 expression in stage II and III colorectal cancer: results from a population‐based series and a randomized phase III study on adjuvant chemotherapy
Source: Cancer Med. 2016 Jun 8;5(8):1840–9. doi: 10.1002/cam4.766 (PMC4971912; doi:10.1002/cam4.766)
Supplement: Supplementary file 4 — Table S1. Complete scoring results of S100A4 immunohistochemistry in study cohort 1. Table S2. Associations between S100A4 expression and clinicopathological parameters in study cohort 1. Table S3. Associations between S100A4 expression and clinicopathological parameters in study cohort 2. [file CAM4-5-1840-s004.doc]

Supporting Information Table 1. Complete scoring results of S100A4 immunohistochemistry in study cohort 1.

|  | **Nuclear*** | **Cytoplasmic*** |
| --- | --- | --- |
| **Percentage** |  |  |
| 0 % | 78 (10) | 96 (12) |
| <1% | 55 (7) | 12 (2) |
| 1-10% | 119 (15) | 23 (3) |
| 11-33% | 183 (23) | 42 (5) |
| 34-66% | 179 (23) | 29 (4) |
| >66% | 169 (22) | 561 (74) |
| **Intensity** |  |  |
| Negative | 78 (10) | 96 (12) |
| Weak | 390 (50) | 326 (42) |
| Moderate | 163 (21) | 257 (33) |
| Strong | 152 (19) | 104 (13) |

*The number of cases and percentages (in parentheses) is shown

Supporting Information Table 2. Associations between S100A4 expression and clinicopathological parameters in study cohort 1.

|  |  | **S100A4 nuclear** | | **S100A4 cytoplasmic** | |
| --- | --- | --- | --- | --- | --- |
|  |  | **Positive*** | ***P* value^§^** | **Positive*** | ***P* value^§^** |
| **Age** |  |  | 0.24 |  | 0.49 |
| **Gender** |  |  | 0.04 |  | 0.11 |
|  | Female | 67 (16) |  | 171 (41) |  |
|  | Male | 81 (22) |  | 173 (47) |  |
| **TNM stage** |  |  | 0.06 |  | 0.74 |
|  | I | 12 (10) |  | 46 (39) |  |
|  | II | 63 (20) |  | 144 (45) |  |
|  | III | 43 (22) |  | 91 (46) |  |
|  | IV | 30 (20) |  | 62 (42) |  |
|  | ND | 1 |  | 1 |  |
| **pT** |  |  | 0.03 |  | 0.86 |
|  | 1 | 4 (13) |  | 16 (53) |  |
|  | 2 | 12 (11) |  | 43 (39) |  |
|  | 3 | 114 (20) |  | 248 (44) |  |
|  | 4 | 18 (22) |  | 37 (46) |  |
| **pN** |  |  | 0.59 |  | 0.42 |
|  | 0 | 85 (18) |  | 213 (44) |  |
|  | 1 | 49 (23) |  | 98 (46) |  |
|  | 2 | 13 (16) |  | 29 (36) |  |
|  | ND | 7 |  | 7 |  |
| **Differentiation** |  |  | 0.04 |  | 0.49 |
|  | Well | 9 (12) |  | 31 (41) |  |
|  | Moderate | 112 (19) |  | 254 (43) |  |
|  | Poor | 23 (25) |  | 43 (46) |  |
|  | ND | 27 |  | 27 |  |
| **Tumor localization** |  |  | 0.78 |  | 0.11 |
|  | Colon | 108 (18) |  | 247 (42) |  |
|  | Rectum | 39 (21) |  | 95 (50) |  |
|  | Synchronous | 1 (20) |  | 2 (40) |  |

*The number of positive cases and percentages (in parentheses) is shown

**^§^***P* value calculated by Fisher´s exact test, linear by linear association chi square test or independent samples t-test as appropriate

ND = not determined

Supporting Information Table 3. Associations between S100A4 expression and clinicopathological parameters in study cohort 2.

|  |  | **S100A4 nuclear** | | **S100A4 cytoplasmic** | |
| --- | --- | --- | --- | --- | --- |
|  |  | **Positive*** | ***P* value^§^** | **Positive*** | ***P* value^§^** |
| **Age** |  |  | 0.87 |  | 0.59 |
| **Gender** |  |  | 0.45 |  | 0.90 |
|  | Female | 42 (23) |  | 42 (23) |  |
|  | Male | 38 (20) |  | 43 (23) |  |
| **TNM stage** |  |  | 0.001 |  | 0.13 |
|  | II | 34 (16) |  | 44 (20) |  |
|  | III | 46 (31) |  | 41 (27) |  |
| **pT** |  |  | 0.06 |  | 0.66 |
|  | 1 | 1 (33) |  | 1 (33) |  |
|  | 2 | 2 (7) |  | 5 (18) |  |
|  | 3 | 69 (22) |  | 73 (23) |  |
|  | 4 | 8 (35) |  | 6 (26) |  |
| **pN** |  |  | 0.001 |  | 0.09 |
|  | 0 | 34 (16) |  | 44 (20) |  |
|  | 1 | 27 (27) |  | 25 (25) |  |
|  | 2 | 15 (34) |  | 14 (32) |  |
|  | 3 | 4 (57) |  | 2 (29) |  |
| **Differentiation** |  |  | 0.30 |  | 0.73 |
|  | Well/moderate | 64 (21) |  | 68 (22) |  |
|  | Poor | 16 (28) |  | 14 (24) |  |
|  | ND | 6 |  | 6 |  |
| **Tumor localization** |  |  | 0.58 |  | 0.79 |
|  | Colon | 59 (23) |  | 59 (23) |  |
|  | Rectum | 21 (19) |  | 26 (24) |  |

*The number of positive cases and percentages (in parentheses) is shown

**^§^***P* value calculated by Fisher´s exact test, linear by linear association chi square test or independent samples t-test as appropriate

ND = not determined
